# Supplementary material for: Altered central pain processing in fibromyalgia—A multimodal neuroimaging case-control study using arterial spin labelling
Source: PLoS One. 2021 Feb 2;16(2):e0235879. doi: 10.1371/journal.pone.0235879 (PMC7853499; doi:10.1371/journal.pone.0235879)
Supplement: S1 Fig — $ according to criteria of American College of Rheumatology; *patients with light opioids, antidepressants, pregabalin or gabapentin included; °5 patients with mental retardation, 3 patients with pregnancy. (DOCX) [file pone.0235879.s001.docx]

S1 Fig: Flow diagram of fibromyalgia patients and controls clinically evaluated between 1^st^ July 2011 and 30^th^ June 2013 and recruited for the study between 1^st^ November 2013 and 31^th^ January 2015 at the University Hospital of Bern. ^$^ according to criteria of American College of Rheumatology; *patients with light opioids, antidepressants, pregabalin or gabapentin included; °5 patients with mental retardation, 3 patients with pregnancy.

**Control pool** **N=9253**

**Fibromyalgia screened N=151**

**Fibromyalgia pool** **N=238**

**Controls screened N=418**

Random sampling

Random sampling

**Controls excluded N=247**

**Fibromyalgia excluded N=77**

Not pain-free N=73 N=6

Fibromyalgia diagnosis not confirmed^$^

- WPI and SSS too low N=11

- other rheumatologic diseases N=12

Left or ambidextrous handedness N=9

MRI interference N=10

Left or ambidextrous handedness N=6

Neurologic or psychiatric co-morbidity

- neurologic N=41

- psychiatric N=45

MRI interference N=9

Neurologic or psychiatric co-morbidity

- neurologic N=11

- psychiatric N=22

Severe somatic co-morbidity N=44

Centrally acting drugs* N=6

Severe somatic co-morbidity N=1

Language problems N=11

Centrally acting drugs* N=3

Other° N=8

Language problems N=2

**Controls eligible N=171**

**Fibromyalgia eligible N=74**

Denied N=59

Inability to contact N=80

Denied N=33

Inability to contact N=9

**Controls included N=32**

**Fibromyalgia included N=32**
