# Supplementary material for: Altered central pain processing in fibromyalgia—A multimodal neuroimaging case-control study using arterial spin labelling
Source: PLoS One. 2021 Feb 2;16(2):e0235879. doi: 10.1371/journal.pone.0235879 (PMC7853499; doi:10.1371/journal.pone.0235879)
Supplement: S1 Table — Results are data-based mean rsCBF with corresponding standard deviation (SD) and model-based unadjusted mean differences of rsCBF with corresponding 95% confidence intervals (CI), t-values and p-values from multivariable general linear models. (DOCX) [file pone.0235879.s002.docx]

S1 Table: Unadjusted differences in resting state perfusion (rsCBF, ml/100g/min) between 32 fibromyalgia patients and 32 pain-free controls in 11 pre-specified Regions of Interest. Results are data-based mean rsCBF with corresponding standard deviation (SD) and model-based adjusted mean differences of rsCBF with corresponding 95% confidence intervals (95% CI), t-values and p-values from multivariable general linear models.

|  | | |  |  |  |  |  |  |  |  |  |
| --- | --- | --- | --- | --- | --- | --- | --- | --- | --- | --- | --- |
| **MNI-coordinates** | | | **Brain area*** |  | **Mean rsCBF (SD)**  **fibromyalgia patients** | **Mean rsCBF (SD)**  **pain-free controls** |  | **Unadjusted mean difference rsCBF (95% CI)** | **T-value** | **p-**  **uncorr** | **P_FWE_** |
| x | y | z |  |  |  |  |  |  |  |  |  |
| -48 | -27 | 24 | L insula |  | 36.65 (11.27) | 36.07 (11.12) |  | -1.52 (-6.80, 3.77) | -0.57 | 0.57 | 1.0 |
| 39 | 4 | 1 | R insula |  | 45.06 (13.63) | 43.43 (12.19) |  | -1.19 (-7.07, 4.70) | -0.40 | 0.69 | 1.0 |
| 43 | -2 | 1 | R insula |  | 45.74 (13.25) | 41.89 (14.70) |  | 1.09 (-5.44, 7.62) | 0.33 | 0.74 | 1.0 |
| -46 | -12 | 5 | L STG/insula |  | 52.60 (17.43) | 47.69 (14.60) |  | 2.48 (-5.37, 10.34) | 0.63 | 0.53 | 1.0 |
| 56 | -23 | 5 | R STG |  | 52.88 (12.50) | 50.46 (12.16) |  | -0.57 (-5.98, 4.84) | -0.21 | 0.83 | 1.0 |
| -15 | -48 | 72 | L SI |  | 31.07 (19.80) | 35.97 (16.49) |  | 5.27 (-12.30, 22.83) | 0.60 | 0.55 | 1.0 |
| -62 | -25 | 17 | L SII |  | 43.96 (11.01) | 42.49 (12.67) |  | -1.31 (-6.58, 3.97) | -0.50 | 0.62 | 1.0 |
| -2 | 48 | -12 | L ACC |  | 47.79 (15.15) | 45.16 (13.26) |  | -8.33 (-16.92, 0.26) | -1.94 | 0.06 | 0.66 |
| -17 | 30 | 31 | L MCC |  | 45.06 (13.63) | 43.43 (12.19) |  | -1.30 (-6.90, 4.30) | -0.46 | 0.64 | 1.0 |
| 13 | -55 | 7 | R lingual gyrus |  | 52.79 (13.08) | 47.92 (10.38) |  | 1.69 (-3.25, 6.64) | 0.69 | 0.50 | 1.0 |
| 27 | -12 | -15 | R amygdala |  | 27.87 (10.07) | 27.39 (12.76) |  | 0.57 (-6.42, 7.56) | 0.16 | 0.87 | 1.0 |
| * ROI selection based on Dehghan, M., et al., Coordinate-based (ALE) meta-analysis of brain activation in patients with fibromyalgia. Hum Brain Mapp, 2016. 37(5): p. 1749-58 with 27 voxels (162mm^3^) boxes centered on MNI-coordinates  Positive unadjusted mean differences suggest increased regional rsCBF in patients as compared to controls after controlling for mean global rsCBF,  MNI: Montreal Neurological Institute coordinates  L: left, R: right  FWE: Family Wise Error correction for multiple comparison  STG: superior temporal gyrus, SI: primary sensory cortex, SII: secondary sensory cortex, ACC: anterior cingulate cortex, MCC: middle cingulate cortex | | | | | | | | | | | |
