# Supplementary material for: Altered central pain processing in fibromyalgia—A multimodal neuroimaging case-control study using arterial spin labelling
Source: PLoS One. 2021 Feb 2;16(2):e0235879. doi: 10.1371/journal.pone.0235879 (PMC7853499; doi:10.1371/journal.pone.0235879)
Supplement: S2 Table — Results are data-based mean GMV with corresponding standard deviation (SD) and model-based unadjusted mean differences of GMV with corresponding 95% confidence intervals (CI), t-values and p-values from multivariable general linear models. (DOCX) [file pone.0235879.s003.docx]

S2 Table: . Adjusted differences in grey matter volume between (GMV) 32 fibromyalgia patients and 32 pain-free controls in 10 pre-specified Regions of Interest. Results are data-based mean GMV with corresponding standard deviation (SD) and model-based adjusted mean differences of GMV with corresponding 95% confidence intervals (CI), t-values and p-values from multivariable general linear models.

|  | | |  |  | |  |  |  |  |  |  |
| --- | --- | --- | --- | --- | --- | --- | --- | --- | --- | --- | --- |
| **MNI-coordinates** | | | **Brain area*** | **Mean GMV (SD)**  **fibromyalgia patients** | | **Mean GMV (SD)**  **pain-free controls** |  | **Unadjusted mean difference GMD (95% CI)** | **T-value** | **P-**  **uncorr** | **P_FWE_** |
| x | y | z |  |  |  |  |  |  |  |  |  |
| -35 | 5 | 2 | L insula |  | 0.44 (0.05) | 0.45 (0.05) |  | 0.00 (-0.02, 0.02) | -0.09 | 0.93 | 1.0 |
| 39 | 5 | 1 | R insula |  | 0.43 (0.06) | 0.44 (0.05) |  | 0.00 (-0.03, 0.02) | -0.21 | 0.84 | 1.0 |
| -53 | -22 | 6 | L STG |  | 0.37 (0.04) | 0.38 (0.04) |  | -0.01 (-0.02, 0.01) | -0.53 | 0.60 | 1.0 |
| 58 | -23 | 5 | R STG |  | 0.35 (0.05) | 0.36 (0.04) |  | -0.01 (-0.02, 0.01) | -0.70 | 0.49 | 1.0 |
| -43 | -24 | 48 | L postcentral gyrus (SI) |  | 0.29 (0.03) | 0.30 (0.04) |  | -0.01 (-0.02, 0.01) | -0.56 | 0.58 | 1.0 |
| -47 | -10 | 13 | L rolandic operculum (SII) |  | 0.39 (0.05) | 0.40 (0.05) |  | -0.01 (-0.03, 0.02) | -0.55 | 0.58 | 1.0 |
| -4 | 34 | 13 | L ACC |  | 0.38 (0.05) | 0.39 (0.04) |  | 0.00 (-0.02, 0.02) | -0.34 | 0.74 | 1.0 |
| -6 | -16 | 40 | L MCC |  | 0.39 (0.05) | 0.40 (0.04) |  | 0.00 (-0.01, 0.02) | 0.46 | 0.65 | 1.0 |
| 16 | -68 | -5 | R lingual gyrus |  | 0.40 (0.04) | 0.42 (0.05) |  | 0.00 (-0.02, 0.01) | -0.55 | 0.59 | 1.0 |
| 27 | -1 | -19 | R amygdala |  | 0.51 (0.04) | 0.52 (0.04) |  | -0.01 (-0.02, 0.01) | -1.13 | 0.26 | 1.0 |
| * ROI selection based on Tzourio-Mazoyer, N., et al., Automated anatomical labeling of activations in SPM using a macroscopic anatomical parcellation of the MNI MRI single-subject brain. Neuroimage, 2002. 15(1): p. 273-89 Provided coordinates are the centers of mass of the ROIs.  Negative unadjusted mean differences suggest decreased GMD in patients as compared to controls after controlling for total intracranial  MNI: Montreal Neurological Institute coordinates. Coordinates are centres of mass.  FWE: Family Wise Error correction for multiple comparison  L: left, R: right, STG: superior temporal gyrus, SI: primary sensory cortex, SII: secondary sensory cortex, ACC: anterior cingulate cortex, MCC: middle cingulate cortex | | | | | | | | | | | |
