# Supplementary material for: Altered central pain processing in fibromyalgia—A multimodal neuroimaging case-control study using arterial spin labelling
Source: PLoS One. 2021 Feb 2;16(2):e0235879. doi: 10.1371/journal.pone.0235879 (PMC7853499; doi:10.1371/journal.pone.0235879)
Supplement: S3 Table — Results are data-based mean CoTh with corresponding standard deviation (SD) and model-based unadjusted mean differences of CoTh with corresponding 95% confidence intervals (CI), t-values and p-values from multivariable general linear models. (DOCX) [file pone.0235879.s004.docx]

S3 Table: Unadjusted differences in cortical thickness (CoTh, mm) between 32 fibromyalgia patients and 32 pain-free controls in 23 pre-specified Regions of Interest. Results are data-based mean CoTh with corresponding standard deviation (SD) and model-based mean differences of CoTh with corresponding 95% confidence intervals (CI), t-values and p-values from multivariable general linear models.

| **Brain Region*** | | **Mean CoTh (SD)**  **fibromyalgia patients** | **Mean CoTh (SD)**  **pain-free controls** |  | **Unadjusted mean difference CoTh (95% CI)** | **T-value** | **p-**  **uncorr** | **P_FWE_** |
| --- | --- | --- | --- | --- | --- | --- | --- | --- |
|  |  |  |  |  |  |  |  |  |
| L insula | L insula long G and S centralis | 3.15 (0.28) | 3.28 (0.29) |  | -0.14 (-0.29, 0.00) | -2.02 | 0.05 | 1.0 |
|  | L insula short G | 3.56 (0.24) | 3.60 (0.27) |  | -0.04 (-0.17, 0.09) | -0.67 | 0.51 | 1.0 |
|  | L insula anterior circular S | 2.72 (0.24) | 2.72 (0.23) |  | 0.01 (-0.11, 0.13) | 0.12 | 0.91 | 1.0 |
|  | L insula inferior circular S | 2.69 (0.19) | 2.75 (0.19) |  | -0.04 (-0.13, 0.05) | -0.90 | 0.37 | 1.0 |
|  | L insula superior circular S | 2.44 (0.17) | 2.43 (0.14) |  | 0.02 (-0.06, 0.10) | 0.39 | 0.70 | 1.0 |
| R insula | R insula long G and S centralis | 3.35 (0.35) | 3.43 (0.29) |  | -0.08 (-0.24, 0.09) | -0.96 | 0.34 | 1.0 |
|  | R insula short G | 3.46 (0.20) | 3.52 (0.20) |  | -0.07 (-0.17, 0.03) | -1.37 | 0.18 | 1.0 |
|  | R insula anterior circular S | 2.69 (0.22) | 2.74 (0.26) |  | -0.05 (-0.17, 0.08) | -0.76 | 0.45 | 1.0 |
|  | R insula inferior circular S | 2.67 (0.23) | 2.63 (0.19) |  | 0.05 (-0.06, 0.16) | 0.88 | 0.38 | 1.0 |
|  | R insula superior circular S | 2.46 (0.14) | 2.45 (0.13) |  | 0.01 (-0.06, 0.08) | 0.34 | 0.74 | 1.0 |
| L STG | L transversal STG | 2.31 (0.21) | 2.36 (0.20) |  | -0.06 (-0.16, 0.05) | -1.09 | 0.28 | 1.0 |
|  | L lateral STG | 2.98 (0.20) | 3.02 (0.22) |  | -0.03 (-0.14, 0.08) | -0.58 | 0.56 | 1.0 |
|  | L STG planum polare | 3.39 (0.31) | 3.44 (0.25) |  | -0.02 (-0.16, 0.11) | -0.34 | 0.74 | 1.0 |
|  | L STG planum temporale | 2.42 (0.26) | 2.52 (0.23) |  | -0.10 (-0.22, 0.03) | -1.51 | 0.14 | 1.0 |
| R STG | R transversal STG | 2.35 (0.24) | 2.43 (0.21) |  | -0.08 (-0.19, 0.04) | -1.38 | 0.17 | 1.0 |
|  | R lateral STG | 2.98 (0.22) | 3.01 (0.22) |  | -0.02 (-0.13, 0.09) | -0.41 | 0.69 | 1.0 |
|  | R STG planum polare | 3.28 (0.28) | 3.27 (0.25) |  | 0.04 (-0.09, 0.16) | 0.56 | 0.58 | 1.0 |
|  | R STG planum temporale | 2.51 (0.29) | 2.49 (0.17) |  | 0.03 (-0.09, 0.15) | 0.51 | 0.62 | 1.0 |
| SI | L postcentral G | 2.17 (0.16) | 2.24 (0.18) |  | -0.06 (-0.14, 0.03) | -1.32 | 0.19 | 1.0 |
| SII | L subcentral G and S | 2.60 (0.15) | 2.65 (0.19) |  | -0.04 (-0.12, 0.05) | -0.83 | 0.41 | 1.0 |
|  | L ACC | 2.53 (0.17) | 2.57 (0.22) |  | -0.06 (-0.16, 0.04) | -1.28 | 0.21 | 1.0 |
|  | L MCC | 2.57 (0.18) | 2.58 (0.22) |  | -0.02 (-0.12, 0.08) | -0.38 | 0.71 | 1.0 |
|  | R lingual G | 2.01 (0.13) | 2.02 (0.13) |  | -0.01 (-0.08, 0.06) | -0.34 | 0.73 | 1.0 |
| * ROI selection based on Destrieux, C., et al., Automatic parcellation of human cortical gyri and sulci using standard anatomical nomenclature. Neuroimage, 2010. 53(1): p. 1-15.) No coordinates are provided as this is a surface based analysis on a spherical surface.  Negative unadjusted mean differences suggest decreased CoTh in patients as compared to controls after controlling for total intracranial  FWE: Family Wise Error correction for multiple comparison  L: left, R: right G: gyrus, S: sulcus  STG: superior temporal gyrus, SI: primary sensory cortex, SII: secondary sensory cortex, ACC: anterior cingulate cortex, MCC: middle cingulate cortex | | | | | | | | |
