# Supplementary material for: Altered central pain processing in fibromyalgia—A multimodal neuroimaging case-control study using arterial spin labelling
Source: PLoS One. 2021 Feb 2;16(2):e0235879. doi: 10.1371/journal.pone.0235879 (PMC7853499; doi:10.1371/journal.pone.0235879)
Supplement: Attachment — Submitted filename: Reply to reviewers.docx [file pone.0235879.s005.docx]

**Response to reviewer for manuscript:**

**Altered central pain processing in fibromyalgia – a multimodal neuroimaging case-control study using arterial spin labelling (PONE-D-20-19081)**

Dear Professor Sommer

Thank you and the editorial team for giving us the opportunity to revise our manuscript. We thank the reviewers for their valuable comments, which we address point-by-point. Our responses are indented and can be found after the corresponding comment. Extracts from the manuscript are in italics and changes in the manuscript are highlighted in red. We believe that our manuscript has greatly benefitted from the feed-back of the two reviewers.

Yours sincerely

Prof. Michele Curatolo

**Reviewer #1:** 

In the article "Altered central pain processing in fibromyalgia - a multimodal neuroimaging case-control study using arterial spin labeling" the authors use classical T1 MRI imaging, arterial spin labeling (ASL) and functional BOLD brain connectivity analysis to compare fibromyalgia patients with healthy controls without a specific stimulus paradigm. They also use questionnaires to investigate the severity of the core symptoms of fibromyalgia (FMS). All investigations have already been published on FMS patients and controls. There are not so many publications on ASL, but there are a lot on rs-fMRI and structural cortex measurements. A positive feature of the study is that they publish null findings, which is in contrast to the usual publications in the structural and connectivity area. In contrast to these studies, their number of subjects (32 per group) is relatively high (upper average) and the authors correct their statistical results, except for the correlation analyses, by multiple comparisons (FWE or FDR). Furthermore, in their ROI analyses they selected the ROIs by a-priori hypotheses of a previous meta-analysis. The methods used are in accordance with current standards in neuroimaging and support the statements in the results section.

Authors reply: Thank you very much for your careful review and the positive evaluation. With your comment #17 you pointed out an error from our side. To avoid misunderstandings while addressing the following comments, we would like to address the error at this place. We analyzed grey matter volume rather than grey matter density. Grey matter volume (GMV) is defined as the proportion of grey matter in the corresponding area, taking into account the size and shape of the brain. We clarified this in the methods section and changed the term grey matter density to grey matter volume throughout the manuscript and in all reviewer comments.

**1.** 71 and elsewhere: These quotations are from older publications, there are now more recent reviews and publications.

Authors reply: This reviewer’s comment is in line with the comment #21 and the reviewers’ general suggestion to include more recent publications. As stated in the introduction our primary objective was to compare rsCBF patterns of fibromyalgia patients and pain-free controls using ASL as an advanced neuroimaging technique providing a direct marker of neural activity at rest. We therefore centered our introduction on evidence of functional markers of pain processing. We agree with the reviewer that taking into account also more recent evidence on BOLD functional connectivity and structural markers should be provided in the discussion. We address this in our reply to the reviewer comment #21. As for the introduction we adapted the sentence the reviewer is referring to as follows:

*“First neuroimaging studies investigating functional markers mainly focused on the comparison of acute pain processing mechanisms in fibromyalgia patients and pain-free controls using experimentally-evoked pain paradigms and blood oxygenation level dependent (BOLD) contrasts (7, 8).”*

We likewise included newer evidence on BOLD resting state functional connectivity:

“Second, even if the researchers acquired resting-state data, they used BOLD contrasts and quantified resting-state connectivity as marker of chronic pain (19, 20) rather than specific regional resting-state neural activity.”

Specifically we added the following references in the introduction and discussion section:

- Čeko M, Frangos E, Gracely J, Richards E, Wang B, Schweinhardt P, et al. Default mode network changes in fibromyalgia patients are largely dependent on current clinical pain. NeuroImage. 2020;216:116877.
- Napadow V, LaCount L, Park K, As-Sanie S, Clauw DJ, Harris RE. Intrinsic brain connectivity in fibromyalgia is associated with chronic pain intensity. Arthritis and rheumatism. 2010;62(8):2545-55.
- Loggia ML, Segerdahl AR, Howard MA, Tracey I. Imaging Clinically Relevant Pain States Using Arterial Spin Labeling. Pain Rep. 2019;4(4).
- Ceko M, Bushnell MC, Fitzcharles M-A, Schweinhardt P. Fibromyalgia interacts with age to change the brain. NeuroImage: Clinical. 2013;3:249-60.
- Feraco P, Nigro S, Passamonti L, Grecucci A, Caligiuri ME, Gagliardo C, et al. Neurochemical Correlates of Brain Atrophy in Fibromyalgia Syndrome: A Magnetic Resonance Spectroscopy and Cortical Thickness Study. Brain Sci. 2020;10(6).
- Shi H, Yuan C, Dai Z, Ma H, Sheng L. Gray matter abnormalities associated with fibromyalgia: A meta-analysis of voxel-based morphometric studies. Semin Arthritis Rheum. 2016;46(3):330-7.

**2.** 81: "as an advanced" + 82 "a direct marker"

Authors reply: As suggested by the reviewer we corrected the sentence in the introduction:

*“Arterial spin labelling (ASL) as an advanced neuroimaging method quantifies resting-state cerebral flow (rsCBF) as a direct marker of neural activity at rest (15).”*

**3.** 105: "confirmed with the diagnosis"

Authors reply: As suggested by the reviewer, we corrected the sentence in the methods section. We likewise addressed American spelling of the word “enrollment” in the same sentence:

*“We included cases if they were confirmed with the diagnosis of fibromyalgia according to the diagnostic criteria of the American College of Rheumatology at repeat clinical examination at enrollment (2).”*

**4.** 110: An explanation why unipolar depressions were included would be useful here.

Authors reply: As correctly pointed out by the reviewer in his comment #17, depression is considered as one important feature of the Symptom Severity Scale, which, together with the Widespread Pain Index, form the basis of the diagnostic criteria of fibromyalgia according to the American College of Rheumatology. Depression is a very common feature of fibromyalgia and excluding patients with depression would yield a non-representative sample. We therefore felt it would be methodologically inconsistent to exclude patients with unipolar depression. However, unlike previous studies, we included severity of depression as measured by the Beck-Depression Inventory version 2 in all adjusted analyses as co-variable. To address this reviewer’s comment, we included the following paragraph in the methods section “participants and study design” as follows:

*“Their diagnostic criteria are based on the Widespread Pain Index, which characterizes spread of pain, and the Symptom Severity Scale, which assesses different core symptoms of fibromyalgia including depression that is frequently encountered in these patients. We therefore did not exclude patients with co-morbid depression from the study but adjusted our analyses for severity of depression measured by the Beck-Depression Inventory version 2 as described in the statistical section.”*

**5.** Furthermore, it should be described in more detail what is meant by end-stage somatic comorbidity, cardiovascular diseases can also have an influence on functional and structural brain markers.

Authors reply: We agree with the reviewer that the term end-stage somatic comorbidity is unclear. We defined end-stage as untreatable disease that represents a threaten for life in the next few months; this was based on clinical judgment. The reason for this exclusion criteria was mainly that we recruited pain-free individuals through the emergency department of the University Hospital of Bern and the pool of eligible patients at this tertiary care facility included severely comorbid patients. We wanted the pool of pain-free controls to be similar in terms of somatic co-morbidity as the pool of cases to ensure comparability. This is also reflected in the flow chart of the supplements which shows that one fibromyalgia patient was excluded due to these criteria versus 44 controls. We specified this statement in the methods section.

*“Common exclusion criteria for cases and controls were conditions interfering with the MRI acquisition; neurologic co-morbidity or history of neurosurgical intervention; psychiatric co-morbidity other than unipolar depressive disorder; end-stage somatic co-morbidity defined as untreatable disease that represents a threaten for life in the next few months; intake of strong opioids or any psychopharmacological treatment other than anticonvulsants or antidepressants; inability to understand the consequences of study participation; and pregnancy.”*

**6.** 166: Was this preprocessing also done with freesurfer? Structural preprocessing and cortex analysis as well as GMD are mixed up in this paragraph, easier to understand would be to summarize all structural analyses.

Authors reply: As suggested by the reviewer we have re-structured the paragraph “Image preprocessing and calculation of functional and structural neuroimaging markers” and described all structural analyses including exact references for statistical analysis packages. The first paragraph of the preprocessing section reads as follows:

*“We performed structural preprocessing for rsCBF, and GMV analyses in SPM12 (Revision 6225, Welcome Trust, London, U.K.,* [*https://www.fil.ion.ucl.ac.uk/spm/)*](https://www.fil.ion.ucl.ac.uk/spm/))*. T1* images were segmented into gray matter, white matter and cerebrospinal fluid, normalized to the Montreal Neurological Institute (MNI) space and smoothed using an 8 mm full-width at half maximum (FWHM) Gaussian kernel.* *To compute GMV, defined as relative proportion of grey matter in a region, accounting for brain size and shape, we applied voxel-based morphometry in SPM12 to the preprocessed T1* images applying an absolute threshold of 0.2 to the modulated grey matter segmentations. We calculated the total intracranial volume by adding the segmented grey matter, white matter and cerebrospinal fluid volumes. We carried out preprocessing for CoTh analyses using the standard FreeSurfer pipeline for cortical reconstruction 5.3.0 (Laboratory for Computational Neuroimaging at the Athinoula A. Martinos Center for Biomedical Imaging, Boston, U.S.A.,* [*http://surfer.nmr.mgh.harvard.edu/*](http://surfer.nmr.mgh.harvard.edu/)*). This included automatic motion correction, segmentation, intensity normalization, inflation and registration to a spherical atlas.”*

**7.** 187: Here it should be justified why no fieldmaps were taken up to be able to calculate magnetic field inhomogeneities.

Authors reply: We agree with the reviewer that it would have been favorable to acquire fieldmaps to increase signal quality. Total scanning time for each subject was 60min as we included also fiber tracking sequences which we will report in a separate paper. We felt that to extend scanning time needed to acquire fieldmaps for fibromyalgia patients who suffer from chronic pain would have added considerable burden to study participants and could have led to motion of the patients during scanning, resulting in overall decreased image quality. However, we believe that accounting for field inhomogeneity would not have changed the interpretation of our findings. Signal quality would have improved in both groups, the cases and controls alike and a more sensitive method would only have detected small group differences. The relevance of these small effect sizes, if at all present, would be equivocal.

To address this reviewer’s comment we included the following statement in the discussion:

“*Moreover, we did not acquire any fieldmaps and therefore could not correct our analyses for magnetic field inhomogeneity. However, we believe that correcting our analyses for field inhomogeneity would not have changed* *the interpretation of our findings. Signal quality would have improved in both groups, the cases and controls alike and a more sensitive method would only have detected small group differences. The relevance of these small effect sizes, if at all present, would be equivocal*.”

**8.** 189: Here it is unclear whether the classical CONN-ROIs were used or extra ROIs of the Deghan et al publication were implemented.

Authors reply: We did not use the classical pre-registered CONN-ROIs. As described in our methods section, we pre-specified ROIs for functional analyses based on the meta-analysis of Dehghan et al and for structural analyses based on atlases. We clarified this as follows in the methods section.

*“ Even though our main statistical approach was to perform voxel- and vertex-wise whole-brain analyses, we also investigated several pre-specified cortical Regions of Interests (ROIs) likely to be involved in pain processing in fibromyalgia based on previous evidence for functional neuroimaging markers and based on atlases for structural markers. We did not investigate software specific ROIs.”*

**9.** 205: Were these clinical parameters included in the model as statistical co-variables, or should "adjusted" be defined as such?

Authors reply: To address this and comment #15 of this reviewer we renamed “crude analyses” as “unadjusted analyses” and changed it throughout the manuscript including the display items. Unadjusted whole-brain and ROI analyses included only global mean rsCBF (rsCBF) and total intracranial volume (GMV, CoTh) as co-variable. Unadjusted analyses of rsFC did not include any co-variable. Adjusted whole-brain and ROI analyses for all neuroimaging outcomes additionally included BDI-ll and STAI-T to account for the effect of depression and anxiety. To enhance clarity which co-variables were included in unadjusted and adjusted analyses we adapted the statistical analyses section. As for the display items we listed all co-variables that were included in the models either in title of footnotes. The statistical section reads now as follows:

*“We considered disease status (fibromyalgia patients vs pain-free controls) as primary explanatory variable. When modelling the unadjusted group difference of rsCBF we included global mean rsCBF of grey matter as co-variable. Similarly, we included total intracranial volume as co-variable when modelling unadjusted group differences of GMV and CoTh. To account for the effects of depression and anxiety we also modelled adjusted group differences of all neuroimaging markers and additionally included BDI-II and STAI-Trait t-value as co-variables. We followed this approach all whole-brain and ROI analyses of rsCBF, GMV and CoTh including the sensitivity analysis described below. We explored group differences in rsFC and calculated adjusted group differences of rsFC for each pair of the 11 pre-specified ROIs and compared rsFC between patients and controls for all resulting 55 connections. Again, we included BDI-ll and STAI-Trait t-values as co-variables to account for the effects of depression and anxiety.”*

**10.** The correct citations to the analytical programs should be added in the methodological paragraph.

Authors reply: We added the citations of the statistical programs used for each analytical step throughout the methods section.

*“We extracted rsCBF and grey matter values in for ROIs using MarsBaR (Version 0.44,* [*http://marsbar.sourceforge.net/*](http://marsbar.sourceforge.net/)*). “*

*“We performed structural preprocessing for rsCBF, and GMV analyses in SPM12 (Revision 6225, Welcome Trust, London, U.K.,* [*https://www.fil.ion.ucl.ac.uk/spm/)*](https://www.fil.ion.ucl.ac.uk/spm/))*.”*

*“To compute GMV, defined as relative proportion of grey matter in a region, accounting for brain size and shape, we applied voxel-based morphometry in SPM12 to the preprocessed T1* images applying an absolute threshold of 0.2 to the modulated grey matter segmentations.”*

*“We carried out preprocessing for CoTh analyses using the standard FreeSurfer pipeline for cortical reconstruction 5.3.0 (Laboratory for Computational Neuroimaging at the Athinoula A. Martinos Center for Biomedical Imaging, Boston, U.S.A.,* [*http://surfer.nmr.mgh.harvard.edu/)*](http://surfer.nmr.mgh.harvard.edu/))*.”*

*“Preprocessing of rsCBF maps was performed in SPM12 and Matlab (MATLAB 2015a; The MathWorks, Inc., Natick, MA, USA).”*

*“BOLD images were co-registered to the corresponding raw T1* image and then processed using the standard processing pipeline in CONN (Version 15,* [*http://www.nitrc.org/projects/conn)*](http://www.nitrc.org/projects/conn))*.”*

**11.** 241: Were these drugs also taken on the day of the MRI examination?

Authors reply: The patients also took the drugs on the day of the MRI examination. We clarified this by including the following statement:

*“The patients took these drugs also on the day of neuroimaging.”*

**12.** Table 1: Are all results distributed normally, so the mean value is appropriate?

Authors reply: Yes, the variables were distributed normally.

**13.** 255: The DLPFC does not seem to be in the Dehghan regions, in this paragraph a clearer distinction should be made between ROI and whole brain analysis.

Authors reply: To address this reviewer’s comment we included another sub-heading so that whole brain and ROI analyses are better distinguished. The two titles are

*“****Differences in whole brain resting-state cerebral blood flow and correlation with pain characteristics****” and “****Differences in local resting state cerebral blood flow of 11 pre-specified ROIs****”*

**14.** 261: According to the table there are 10 regions which have a lower mean regional blood flow in fibromyalgia patients, is the mean difference meant here?

Authors reply: We think there is a misunderstanding. Table 2 shows, as indicated in the results section, that model-based adjusted mean differences were negative in 3 of the 11 pre-specified ROIs after controlling for global rsCBF, depression and anxiety. This would suggest decreased regional rsCBF in patients as compared to controls in only 3 regions. Likewise as shown in table 2 displaying mean rsCBF in patients and controls, regional blood flow is typically slightly higher in patients.

To clearly distinguish data-based mean rsCBF in cases and controls and model-based estimates of adjusted mean difference after controlling for global mean rsCBF, depression and anxiety we adapted the results section and the title of table 2 accordingly. We also included a clear statement in the footnote of the table to help the reader interpreting positive adjusted mean differences.

*“Although in more than half of the areas (7 of 11 ROIs) model-based adjusted mean difference between patients and controls suggest an increased regional blood flow in patients, none of the increases was statistically significant neither without nor with FWE correction.”*

*“Title of table 2: …. Results are data-based mean rsCBF with corresponding standard deviation (SD) and model-based adjusted mean differences of local rsCBF with corresponding 95% confidence intervals (CI), t-values and p-values from multivariable general linear models.”*

*“Footnote of table 2: Positive adjusted mean differences suggest increased regional rsCBF in patients as compared to controls after controlling for global rsCBF of grey matter, depression and anxiety.”*

**15.** 297: Up to this paragraph it is still not clear to the reader what is meant by "crude" or adjusted analyses. Are the drug subgroups referred to here, or the clinical parameters?

Authors reply: As suggested by the reviewer in his comment #9 we renamed “crude analyses” as “unadjusted analyses” and changed it throughout the manuscript including the display items. Unadjusted whole-brain and ROI analyses included only global mean rsCBF (rsCBF) and total intracranial volume (GMV, CoTh) as co-variable. Unadjusted analyses of rsFC did not include any co-variable. Adjusted whole-brain and ROI analyses for all neuroimaging outcomes additionally included BDI-ll and STAI-T to account for the effect of depression and anxiety. To enhance clarity which co-variables were included in unadjusted and adjusted analyses, we adapted the statistical analysis section. As for the display items we listed all co-variables that were included in the models either in title of footnotes. The statistical section reads now as follows:

*“We considered disease status (fibromyalgia patients vs pain-free controls) as primary explanatory variable. When modelling the unadjusted group difference of rsCBF we included global mean rsCBF of grey matter as co-variable. Similarly, we included total intracranial volume as co-variable when modelling unadjusted group differences of GMV and CoTh. To account for the effects of depression and anxiety we also modelled adjusted group differences of all neuroimaging markers and additionally included BDI-II and STAI-Trait t-value as co-variables. We followed this approach all whole-brain and ROI analyses of rsCBF, GMV and CoTh including the sensitivity analysis described below. We explored group differences in rsFC and calculated adjusted group differences of rsFC for each pair of the 11 pre-specified ROIs and compared rsFC between patients and controls for all resulting 55 connections. Again, we included BDI-ll and STAI-Trait t-values as co-variables to account for the effects of depression and anxiety.”*

We report the subgroup analysis of patients not taking any centrally acting drugs at the end of the results section and again explicitly state that the adjusted analyses included depression and anxiety along with global mean rsCBF or total intracranial volume as co-variables.

*“Adjusted whole-brain subgroup-analyses, including depression, anxiety, and global mean rsCBF or total intracranial volume as co-variables, of 11 (38%) patients not taking centrally acting drugs and matched controls showed no group differences in rsCBF, GMV and CoTh.”*

**16.** Table 3: In which unit or on which scale is GMD measured? Same question for Table 4 and cortical thickness.

Authors reply: We thank the reviewer for this comment as it pointed out an error from our side. We analyzed grey matter volume rather than grey matter density. Grey matter volume (GMV) is defined as the proportion of grey matter in the respective area, taking into account the size and shape of the brain and as such is without unit. We clarified this in the methods section and changed the term grey matter density to grey matter volume throughout the manuscript.

*“To compute GMV, defined as relative proportion of grey matter in a region, accounting for brain size and shape, we applied voxel-based morphometry in SPM12 to the preprocessed T1* images applying an absolute threshold of 0.2 to the modulated grey matter segmentations.”*

Cortical thickness was measured in mm. As suggested by the reviewer we included this and also the unit of rsCBF in table 4 and 2, respectively.

**17.** 373: Depressive symptoms are, however, one of the core symptoms of FMS patients, if the authors only want to measure chronic pain in the brain, this hypothesis is correct, for the "fibromyalgia signature" one would have to exclude major depressions and then analyze them again.

Authors reply: We included severity of depression as co-variable in all adjusted analyses. This suggests that the lack of group differences in neuroimaging markers between fibromyalgia patients and controls is independent of depression. As suggested by the reviewer we performed subgroup whole-brain analyses of rsCBF, GMV and cortical thickness in patients without major depression defined as BDI-ll <19 (N=15) and corresponding controls (N=15). We included anxiety and global mean rsCBF or total intracranial volume as co-variables. As in the main analyses we did not find any group differences in any of the neuroimaging markers.

**18.** 382: Before the authors give this statement, they should consult Üçeyler et al 2013 and the many following publications on functional and structural loss from the small nerve fibres in some FMS patients.

Authors reply: We agree with the author that our statement he is referring to is too strong and adapted the sentence as follows:

*“Fibromyalgia is characterized by intensive, widespread pain not sufficiently explained by peripheral lesions making it a good model to study the pathophysiology of enhanced central pain mechanisms it also shows high co-morbidity with depression and anxiety.”*

**19.** 392: How large was the proportion of patients without medication?

Authors reply: As indicated in the text describing the results of the sub-group analysis, 11 patients did not take any centrally acting drugs. We added the proportion of patients without medication in brackets.

*“Adjusted whole-brain subgroup-analyses including depression, anxiety, and mean grey matter rsCBF or total intracranial volume as covariables of 11 (34%) patients not taking centrally acting drugs and matched controls showed no group differences in rsCBF, GMV and CoTh.”*

**20.** 405: The authors could check how severely their patients were affected compared to the rest of the worldwide patient population by comparing them with other publications using the FIQ or the NRS scale.

Authors reply: To address this reviewer’s comment we included the following statement in the discussion:

*“Severity of fibromyalgia measures by the FIQ in our patients was similar to patients included in key randomized controlled trials evaluating non-pharmacological interventions (48, 49) and phase III effectiveness trials of different drugs (50, 51).”*

**21.** 406: In the whole discussion there are no studies mentioned, which for example see clear changes in functional connectivity, even with multiple comparisons. The same applies to the structural parameters. Even if other results were found, other studies with the same methods should be mentioned.

Authors reply: We would like to stress that our primary objective was to compare rsCBF patterns of fibromyalgia patients and pain-free controls using ASL as we think the scope of the present article should be kept in mind when giving weight in the discussion of the vast and heterogenous literature on functional and structural neuroimaging findings in fibromyalgia. We restated our objectives at the beginning of the discussion and structured the paragraph “scientific context of our study” according to these objectives.

*“Our primary objective was to compare rsCBF patterns of fibromyalgia patients and pain-free controls using ASL as measure of neural activity at rest and thus likely to reproduce a neuroimaging marker of chronic, stimulus-independent pain.”*

*“Our secondary objective was to compare resting-state functional connectivity between pre-specified areas likely to be involved in pain processing, grey matter volume and cortical thickness between cases and controls assuming a reduction in these neuroimaging markers in fibromyalgia.”*

Concerning the literature on functional connectivity in fibromyalgia patients, we believe that the reviewer is referring to the previous studies investigating resting state functional connectivity in fibromyalgia that investigated functional connectivity within the Default Mode Network or between the Insula and the Default Mode Network (Čeko M, Frangos E, Gracely J, Richards E, Wang B, Schweinhardt P, et al. Default mode network changes in fibromyalgia patients are largely dependent on current clinical pain. NeuroImage. 2020;216:116877. And Napadow V, LaCount L, Park K, As-Sanie S, Clauw DJ, Harris RE. Intrinsic brain connectivity in fibromyalgia is associated with chronic pain intensity. Arthritis and rheumatism. 2010;62(8):2545-55). We included the following statement to discuss the findings of these studies:

*“**With regards to the literature on functional connectivity in fibromyalgia patients, our objective was to compare rsFC between pre-specified areas likely to be involved in pain processing as defined by Dehghan and colleagues (8). Previous studies investigated rsFC of the Default Mode Network. These studies found an increased connectivity within the Default Mode Network and between the Insula and the Default Mode Network (19, 20).”*

With regard to the literature on structural neuroimaging markers we believe that Shi and colleagues published the most recent review on grey matter alterations in fibromyalgia (Shi H, Yuan C, Dai Z, Ma H, Sheng L. Gray matter abnormalities associated with fibromyalgia: A meta-analysis of voxel-based morphometric studies. Semin Arthritis Rheum. 2016;46(3):330-7.) We kindly ask the reviewer to further specify any reference of newer date in case we still missed relevant literature in our updated search. We adapted the last part of the section “Scientific context” and included latest evidence:

*“The most recent meta-analysis investigating GMV showed grey matter decreases in the anterior and posterior cingulate cortex, the medial prefrontal cortex and the parahippocampal gyrus or fusiform cortex. However, the original studies which were included in this meta-analysis and the latest study investigating CoTh typically did not correct for co-morbid depression or for multiple comparisons, and if they did, group differences in neuroimaging markers lost statistical significance.”*

**22.** 415: This statement cannot be supported by the results, because no chronic pain processing regions were found. Rather, as mentioned before, the methodology is not sensitive enough.

Authors reply: We agree that we did not find regions involved in chronic pain processing. Because we have primarily analyzed brain areas involved in the processing of acute pain signals, we propose that these areas are unlikely to be involved in the development and maintenance of chronic, stimulus-independent pain. To clarify this point, we have modified the text as follows:

*“Because we analyzed brain areas involved in the processing of acute pain signals, our results suggest that these areas are unlikely to be involved in the development and maintenance of chronic, stimulus-independent pain.”*

**23** Figure 1: It should be clear in which unit the Colour Bar displays, the same applies to Figure 2.

Authors reply: We agree that it must be clear whether the color coding represents t- or z-values. As mentioned in the figure legends, the color bars represent t-values. We now label the color bar in the figures as well.

**Reviewer #2**

The manuscript “Altered central pain processing …” reports on a multimodal MRI study of 32 fibromyalgia patients compared to 32 matched controls. The investigators analyzed resting state cerebral blood flow by arterial spin labeling, resting state functional connectivity, grey matter density, and cortical thickness. The investigators adjusted their analyses to consider multiple different aspects of their participants, including demographics, mood, and the use of pharmaceutics. The emphasis of the paper is on the novel use of arterial spin labeling, which did not turn out as expected, but what is striking to the reviewer is that, despite careful analysis, the investigators did not find any of the differences that have been reported in the literature for gray matter density, etc. Reviewing the methods, the case ascertainment for fibromyalgia patients seems reasonable, using the 2011 criteria. Measures of pain and mood use appropriate instruments. The 12-channel head coil seems like an older coil compared to the newer 64-channel coils, but such should only affect spatial resolution but not impact the ability to detect altered functional measures. The methods appear generally sound with a substantial sample size that should be able to detect changes in each of the measurements made. Overall, the authors do a very credible job in finding no differences between the groups. These negative results are very important to report – as previous reports of changes in these imaging parameters are frequently used as evidence of the ‘pathology’ of fibromyalgia. Demonstrating that evidence of altered neurological activity at resting that would account for differences in pain perception cannot be readily demonstrated is also one of several observations that undermine the concept of “central sensitization” being the cause of fibromyalgia. These results are important for the field to be aware of. To follow are comments about ways to clarify the methods further.

Authors reply: Thank you very much for your careful review and the positive evaluation. The first reviewer pointed out an error from our side. To avoid misunderstandings while addressing your comments, we would like to address the error at this place. We analyzed grey matter volume rather than grey matter density. Grey matter volume (GMV) is defined as the proportion of grey matter in the respective area, taking into account the size and shape of the brain. We clarified this in the methods section and changed the term grey matter density to grey matter volume throughout the manuscript and in all reviewer comments.

**1.** Lines 154-7: Make it clear what the 11 ROIs are. It isn’t obvious from the list here that there are 11 ROIs. Should inform the reader that there are multiple insula ROIs (one ROI in left insula, and two in right insula) defined by Dehghan.

Authors reply: This point is very well taken by the reviewer and in line with a comment of the first reviewer. As suggested by the reviewer in comment #M15 we also used the same term “pre-specified” throughout the manuscript. We therefore adapted the paragraph “Selection of Region of Interests (ROIs)” as follows:

*“****Selection of Regions of Interests (ROIs)***

*Even though our main statistical approach was to perform voxel- and vertex-wise whole-brain analyses, we also investigated several pre-specified cortical Regions of Interests (ROIs) likely to be involved in pain processing in fibromyalgia based on previous evidence for functional and on atlases for structural neuroimaging markers . We did not investigate software specific ROIs. For functional analyses of rsCBF and rsFC we defined the exact coordinates of 11 ROIs according to a recent meta-analysis by Dehghan et al : Bilateral insula, left anterior and middle cingulate cortex (ACC, MCC), right amygdala, bilateral superior temporal gyrus (STG), right lingual gyrus and left primary and secondary cortex (S I, S II). Notably, the right insula is represented in two ROIs in this selection while all other brain regions are represented by one ROI each. We created a box around the coordinates with size = 27 voxels (216 mm^3^). For structural analyses of GMV and CoTh we extracted exact coordinates of brain regions that represented the above specified regions from existing atlases. This resulted in 10 volumetric ROIs based on the Automatic Anatomical Labeling (AAL)-Atlas for GMV analyses and 23 ROIs based on the Destrieux-Atlas which is based on gyral and sulcal surface for CoTh analyses .”*

**2.** S1 Table: Is the 40 for number of ROIs in the header of S1 Table a mistake? The results seem to show only the same ROIs as for the other analyses. Also, these 40 ROIs are not mentioned in the section entitled “Selection of Region of Interests (ROIs)”.

Authors reply: We thank the reviewer for identifying this mistake. Table S1 shows the same 11 ROIs as Table 2 but with unadjusted mean differences including only mean grey matter rsCBF as co-variable. Also taking into account the comments of the first reviewer, we corrected the header of this table:

*“S1 Table. Unadjusted differences in resting state perfusion (rsCBF) between 32 fibromyalgia patients and 32 pain-free controls in 11 pre-specified Regions of Interest. Results are data-based mean regional rsCBF with corresponding standard deviation (SD) and model-based mean differences of regional rsCBF with corresponding 95% confidence intervals (CI), t-values and p-values from multivariable general linear models.”*

**3.** Line 180-1: Unclear what is meant by “We finally modelled a mean rsCBF map for each subject based on 40 pre-processed maps…”. Explain what the 40 pre-processed maps are and where they came from. We assume these are for each of the selected ROIs.

Authors reply: We acquired 40 pairs of unlabelled and labelled images (=80 images). We calculated a rsCBF map for each of these pairs and averaged these 40 maps to increase the signal noise ratio. Statistical analyses were conducted on these mean maps (1 mean map per subject). We adapted the section on CBF quantification to enhance clarity:

*“Second, a set of 80 functional T2* weighted images using a pseudo-continuous arterial spin labelling sequence (pCASL) which corresponds to 40 pairs of unlabeled and labeled images (30, 31).”*

*“We quantified resting-state cerebral blood flow (rsCBF, ml/100g/min) ….. This resulted in 40 unprocessed rsCBF maps per subject. They were realigned and co-registered to the corresponding raw T1* image and normalized using the deformation matrix of the corresponding T1* image.”*

*“This resulted in 40 pre-processed grey matter rsCBF maps per subject. There was no significant difference between groups in motion and we did not exclude any subject due to excessive motion. To increase the signal-to-noise ratio, we finally modelled a mean rsCBF map for each subject based on the 40 pre-processed maps. We used this mean rsCBF map for further data analysis and computed global mean rsCBF within grey matter per subject based on this mean rsCBF map.”*

**4.** Lines 207-8: It is unclear why FWE correction was used for whole brain analyses at peak rsCBF and for cortical density, but FDR correction was used for ROI analyses. A consistent method for correction for multiple comparisons should be used, otherwise, a justification should be given.

Authors reply: The use of FDR versus FWE correction to address multiple comparison is indeed a matter of ongoing debate even though both methods are likewise accepted. Initially, we had chosen distinct corrections for each type of analysis (whole brain vs. ROI), because of the standards and the number of expected statistical comparisons. As the number of statistical comparisons in whole-brain analyses exceeds the number of comparisons in ROI, we believe that a more conservative correction using FEW was justified. However, we agree, that readers might prefer a single type of correction, which is why we now report all ROI analysis results with FWE correction in close alignment with the whole brain analyses. As expected, our results of the ROI analyses did not change when adapting the correction method.

**5.** Lines 208-9: A cluster size threshold of 10 voxels is not justified. However, this is very low threshold and would bias the results away from the null. As the results were the null, this does not seem to be problematic in regard to the results.

Authors reply: We thank the reviewer for identifying this error from our side. We did not apply any cluster size threshold for the analysis but used this threshold in both figures in order not to display irrelevant signals. We deleted the sentence from the text and added it to the legends of the figures.

**6.** Lines 215-6 state that “We considered all neuroimaging markers with group differences at p ≤ 0.10 in any of the crude or adjusted analyses after correction for multiple comparisons for these correlations.” I believe “for these correlations” should be removed here as it seems you are discussing group differences in imaging results, not correlations.

Authors reply: We agree with the reviewer and removed “for these correlations” in the manuscript.

**7.** Also, your p value is .10 corrected for multiple comparisons, but the tables show uncorrected p values. Explain why this was done or correct this.

Authors reply: This comment is in line with the comment #M16 by the reviewer. We agree with the reviewer that we should not selectively report only uncorrected p-values in all tables. We pre-specified the threshold of p≤0.10 to be selected for the correlation analyses. We did not find any group difference at p≤0.10 (corrected) in any of the ROIs for any of the neuroimaging outcomes and therefore did not calculate any correlations for these outcomes. We amended the tables with the FWE-corrected p-values to better inform the reader. We therefore also refrained from removing the footnote in the tables (see comment #M16).

**8.** Line 216: Using a threshold of .7 for significant correlations seems high. Is there justification for using .7? However, this permissive number again would bias away from the null – which does not seem to be impacting the analysis here.

Authors reply: We agree with the reviewer that the threshold of 0.7 is high. The reason for choosing this threshold was that we wanted to have a strong correlation between clinical pain parameters and any potential group differences in neuroimaging markers to interpret it as relevant with enough confidence. As already mentioned by the reviewer, the correlation coefficients we found were very low and ranged between 0.02 and 0.22. Hence, we propose to leave the interpretation of the strength of the correlations to the informed reader and we now have removed the sentence from the manuscript.

**9.** Define what is meant by “crude”. I suggest using a term other than “crude” as “crude” has negative connotations. Lines 259-60 should state for what the analysis for Table 2 was adjusted. Line 262 should state for what S1 Table was adjusted. Also, line 94 states that, “We adjusted all our analyses for depression and anxiety…” (similarly, lines 205-6), but it is not clear what a “crude” analysis is. Line 210 restates this.

Authors reply: This comment is in line with reviewer comment #10 and #12. We agree with the reviewer that the term “crude” has negative connotations and changed it to unadjusted throughout the manuscript including titles of display items. Unadjusted whole-brain and ROI analyses included only global mean rsCBF (rsCBF) and total intracranial volume (GMV, CoTh) as co-variable. Unadjusted analyses of rsFC did not include any co-variable. Adjusted whole-brain and ROI analyses for all neuroimaging outcomes additionally included BDI-ll and STAI-T to account for the effect of depression and anxiety. To enhance clarity which co-variables were included in unadjusted and adjusted analyses we adapted the statistical analyses section. As for the display items we listed all co-variables that were included in the models either in title of footnotes. The statistical section reads now as follows:

*“We considered disease status (fibromyalgia patients vs pain-free controls) as primary explanatory variable. When modelling the unadjusted group difference of rsCBF we included global mean rsCBF of grey matter as co-variable. Similarly, we included total intracranial volume as co-variable when modelling unadjusted group differences of GMV and CoTh. To account for the effects of depression and anxiety we also modelled adjusted group differences of all neuroimaging markers and additionally included BDI-II and STAI-Trait t-value as co-variables. We followed this approach all whole-brain and ROI analyses of rsCBF, GMV and CoTh including the sensitivity analysis described below. We explored group differences in rsFC and calculated adjusted group differences of rsFC for each pair of the 11 pre-specified ROIs and compared rsFC between patients and controls for all resulting 55 connections. Again, we included BDI-ll and STAI-Trait t-values as co-variables to account for the effects of depression and anxiety.”*

**10.** Specify what variable has been adjusted for rsCBF of grey matter in S1 Table.

Authors reply: Please refer to our answer to reviewer comment #9. We included all co-variables and added a statement how to interpret unadjusted and adjusted mean differences in the footnote of all display items including table S1.

**11.** Tables 2, 3, 4, and S1: The rsCBF mean differences are different from actual numerical differences, but it is not clear why.

Authors reply: This reviewer’s comment is very well taken. The mean differences displayed along with their 95% confidence intervals are estimated by the statistical model. We agree with the reviewer that this should be clarified. We therefore included in all the titles that the means (SD) for both groups are data-based and the mean differences (95% CI) are model-based. For the tables 2,3 and 4 this means that they are adjusted mean differences after accounting for mean global rsCBF (table 2) or total intracranial volume (table 3 and 4) and depression and anxiety. For tables S1, S2, S3 this means that they are unadjusted mean differences after accounting only for mean global rsCBF (table S1) or total intracranial volume (table S2 and S3), respectively. We likewise included a sentence in the footnote of all tables on how to interpret the group differences. We include the new title and the footnote of table 2 and table S1 as an example.

“Table 1: Adjusted differences in resting state perfusion (rsCBF, ml/100g/min) between 32 fibromyalgia patients and 32 pain-free controls in 11 pre-specified Regions of Interest. Results are data-based mean rsCBF with corresponding standard deviation and model-based adjusted mean differences of rsCBF with corresponding 95% confidence intervals (CI), t-values and p-values from multivariable general linear models.”

“Positive adjusted mean differences suggest increased regional rsCBF in patients as compared to controls after controlling for mean global rsCBF, depression and anxiety.”

“Table S1: Unadjusted differences in resting state perfusion (rsCBF, ml/100g/min) between 32 fibromyalgia patients and 32 pain-free controls in 11 pre-specified Regions of Interest. Results are data-based mean rsCBF with corresponding standard deviation and model-based adjusted mean differences of rsCBF with corresponding 95% confidence intervals (CI), t-values and p-values from multivariable general linear models.”

“Positive unadjusted mean differences suggest increased regional rsCBF in patients as compared to controls after controlling for mean global rsCBF”.

**12.** S1 Table: Were the mean differences here adjusted or not? It should say in the header, similar to Table 2.

Authors reply: Please refer to our answer to reviewer comment #10. We included all co-variables and added a statement how to interpret unadjusted and adjusted mean differences in the footnote of all display items including table S1. Likewise, as suggested by the reviewer we explicitly mentioned in the header that the mean differences of table S1 are unadjusted.

**13.** Line 306 (Table 3): What are “adjusted mean differences” here? If they are adjusted for total intracranial volume, depression and anxiety, state that in the header or text. Are the mean GMDs for each patient group adjusted also?

Authors reply: This comment is in line with the previous comments #9-12. The mean GMV for each patient group is data-based and hence not adjusted. The adjusted mean differences means that the results are model-based and controlled for total intracranial volume, depression and anxiety. We clarified these points in the header and footnote of table 3:

“Table 3: Adjusted differences in grey matter volume between (GMV) 32 fibromyalgia patients and 32 pain-free controls in 10 pre-specified Regions of Interest. Results are data-based mean GMV with corresponding standard deviation (SD) and model-based adjusted mean differences of GMV with corresponding 95% confidence intervals (CI), t-values and p-values from multivariable general linear models.”

“Negative adjusted mean differences suggest decreased GMV in patients as compared to controls after controlling for total intracranial volume, depression and anxiety.”

**14.** Headers for Tables 2 and 3 should state that a 3x3x3 voxel box around the MNI coordinates is used as the ROI, not the full brain region. It would be preferable for the mean of the full ROI be used, rather than just the mean of a small box.

Authors reply: As described in the methods section, we created a 27 voxels (216 mm3) space around the MIN coordinates for the functional ROIs of table 2. As rsCBF is a functional neuroimaging marker we did not employ an anatomical-structural definition of the full ROI for table 2. As we suggested by the reviewer, we added this on the food-note of table 2.

*“ *ROI selection based on Dehghan, M., et al. Coordinate-based (ALE) meta-analysis of brain activation in patients with fibromyalgia. Hum Brain Mapp, 2016. 37(5): p. 1749-58. with 27 voxels (216mm^3^) boxes centered on MNI-coordinates.”*

Table 3 however shows results for GMV ROI analyses and we agree with the reviewer that for structural analyses the full ROI which corresponds to the atlas region should be used, approach that we already implemented as described in the methods section. Hence we did not use the 27 voxels (216 mm3) space around the MIN coordinates for table 3.

*“ *ROI selection based on Dehghan, M., et al. Coordinate-based (ALE) meta-analysis of brain activation in patients with fibromyalgia. Hum Brain Mapp, 2016. 37(5): p. 1749-58* Provided coordinates are the centers of mass of the ROIs *.”*

**15.** The greatest weakness of this study is that no measurements of pain perception were made during the scanning sessions. While scores estimating the burden of pain experienced within the last 24 hours before the scan were collected, the investigators cannot state with certainty that the fibromyalgia patients were experiencing clinically relevant pain while MRI measures were made. It also seems that strongest correlative work would relate the sensation occurring at the time of scanning to the scans.

Authors reply: We collected pain ratings immediately before the scan and added them in the baseline table 1. As expected in fibromyalgia patients which typically show widespread, high and constant pain, mean pain immediately before the scan was 6.0 (SD 2.2) as compared to average pain during the 24h before scanning which was 6.2 (SD 1.9). As we measured pain intensity immediately before scanning and fibromyalgia patients do not typically have substantial short-term fluctuations in pain, we believe it would have been unnecessary to repeat pain intensity measures during scanning. However, we now also calculated correlation coefficients between group differences in rsCBF and pain intensity immediately before scanning and again found no relevant correlations. We therefore adapted both figures and amended the methods and results section as follows:

*“In cases we additionally assessed pain intensity, pain duration, spread of pain and disability. We used the Numerical Rating Scale to measure average pain intensity within the last 24 hours, pain intensity immediately before neuroimaging (NRS, 0 = no pain to 10 = worst pain) and the Fibromyalgia Impact Questionnaire to assess disability (FIQ, 0 = no disability, 100 = most severe disability).”*

*“However, rsCBF in these two regions was not correlated with average pain intensity 24h before or pain intensity immediately before neuroimaging spread of pain or disability in 32 fibromyalgia patients (Fig 1 and 2 B).”*

In regard to the discussion, I would opine it can be approved by addressing the comments below:

**16.** What do their ASL findings mean? The authors do note decreased rsCBF in the r dorsolateral PFC and l inferior middle TMG that do not correlate with pain measures. Can these changes be part of fibromyalgia pathology – or do they reflect false positive statistical phenomenon related to performing multiple comparison analyses? Please comment.

Authors reply: This reviewer comment is very well taken. We amended the discussion section as follows:

*“We believe that the decrease in rsCBF in the Dorsolateral Prefrontal Cortex is likely to be an effect of depression as the role of the Dorsolateral Prefrontal Cortex in depression is well established (42, 43) and the effect could not be observed anymore after controlling the analysis for depression. With regard to the decreased rsCBF in the Inferior Middle Temporal Gyrus we propose that this effect may either indicate a process secondary to the pain experience, such as the subjective evaluation of pain, or a false positive statistical finding rather than an effect attributable to pain. The rationale behind this is fourfold. First, there is no prior evidence for a pathophysiological involvement of the Inferior Middle Temporal Gyrus in fibromyalgia. Second, we detected a decreased rsCBF in this area in patients as compared to controls which is contrary to our main hypothesis of increased rsCBF in cases as result of central hypersensitivity. Third, we did not find any relevant correlations between rsCBF in this area and pain characteristics. Fourth, we were unable to see any group differences between cases and controls in this area for any other neuroimaging marker. In view of the large number of analyses conducted, a false positive statistical finding is a possible explanation.”*

**17.** This is the largest study to date to look at rsfunctional connectivity, gray matter volume, and cortical thickness – and perhaps the first to show negative findings this definitively. These negative findings only receive two sentences in the discussion (lines 372-375). Considering that these types of changes reported in smaller and poorly adjusted papers are often cited as evidence of the pathology of fibromyalgia, I opine that the authors should comment on this in the discussion.

Authors reply: We discuss possible explanations of our negative study and more strongly emphasized the methodological advantages of our study as compared to previous publications in the section “strengths and limitations”:

*“Our study included totally 64 participants, making it one of the largest in the field. Therefore, we do not think that our inability to observe statistically significant group differences was merely due to a lack of power. Our results were consistent across four imaging modalities and robust to the type of analysis (whole-brain and ROI-analyses, subgroup analysis in patients not taking centrally acting drugs). Additionally, we were unable to detect relevant correlation between neuroimaging markers with clinically important outcomes such as pain intensity, spread of pain and disability. Still, an argument for limited power to detect relevant group differences is the fact that patients showed numerically increased rsCBF, decreased GMV and decreased CoTh in most of the pre-specified ROIs even after adjusting for depression and anxiety what would be in line with our a priori hypotheses. Previous studies showing group-differences were both smaller and of less methodological rigor as compared to our study. They typically did not account for co-morbid depression and intake of pain-modulating, centrally acting drugs even though both features are particularly frequent in fibromyalgia and likely to influence neuroimaging results.”*

**18.** Add lack of in-MRI pain measurements to the strength and limitations section.

Authors reply: Please refer to the answer of comment # 15.

**19.** The authors may want to comment on the proteus phenomenon (<https://www.ncbi.nlm.nih.gov/pmc/articles/PMC1182327/>) in their discussion about false negatives (lines 400-406).

Authors reply:

*“Furthermore, evidence generation in the field of neuroimaging and fibromyalgia is still in its early stage. This phase of evidence generation is often characterized by conflicting findings as expression of the evidence accumulating process itself, previously referred to as proteus phenomenon (43, 44). Therefore, our inability to find evidence to support the hypothesis of altered central pain processing might also be interpreted in the light of this phenomenon.”*

**20.** Line 409: ‘may point out’ should be ‘demonstrate’.

Authors reply: As suggested by the reviewer we changed the wording accordingly.

Here are some minor comments to consider as well:
Minor comments:

**M1.** I recommend not using “CT” as the abbreviation for “cortical thickness”, since it is more often used to mean “CAT scan”.

Authors reply: As suggested by the reviewer we replaced CT with CoTh in the whole manuscript to avoid misunderstanding.

**M2.** Line 42: Change “as brain” to “as brain areas” or “as regions of interest”.

Authors reply: We thank the reviewer for spotting this error and corrected it accordingly to “as brain areas”

**M3.** Line 165: place “MNI” in parentheses. Use “smoothed” instead of “smoothened”.

Authors reply: We adapted the sentence as suggested by the reviewer.

**M4**. Line 171: “TI2” is defined in the text but it is not used in the equation, so remove it.

Authors reply: Again, we thank the reviewer for spotting this error and corrected it accordingly

**M5.** Lines 229, 231: Change “enrolment” to “enrollment”.

Authors reply: As suggested by the reviewer we now use American spelling throughout the manuscript.

**M6.** Table 1: Don’t use “*” to mean Student’s T test as it is commonly used to signify statistical significance.

Authors reply: We agree with the reviewer and use “^+^” to denote t-tests:

**M7.** Line 256 and elsewhere: Use “voxels” rather than “voxel” to specify the cluster sizes.

Authors reply: We changed “voxel” to “voxels” to specify the cluster size throughout the manuscript.

**M8.** Line 261-2: I suggest changing to wording of “None of the increases was statistically significant neither without nor with FDR correction” to “None of the increases was statistically significant with or without FDR correction”.

Authors reply: We changed the sentence as suggested by the reviewer and and as suggestion by the reviewer’s comment #4 use now FWE throughout the manuscript.

**M9.** Line 265: Figure 1 text: Clean up the wording so as not to repeat descriptions of A) and B). Same for Figure 2 text.

Authors reply: We adjusted both figure headers as follows also in view of consistency with the wording Unadjusted and Adjusted throughout the manuscript and in line with previous comments of the reviewer:

*“Figure 1: Unadjusted difference in resting-state cerebral blood flow (rsCBF) between cases (N=32) and controls (N=32) in the right Dorsolateral Prefrontal Cortex (x=44; y=32; z=26) controlling for mean global rsCBF (A) and correlation of pain characteristics with rsCBF of this region within patients (N=32) (B).”*

*“Figure 2: Adjusted difference in resting-state cerebral blood flow (rsCBF) between cases (N=32) and controls (N=32) in the left Inferior Middle Temporal Gyrus (x=-50; y= -50; z=-4) controlling for mean global rsCBF, depression and anxiety (A) and correlation of pain characteristics with rsCBF of this region within patients (N=32) (B).”*

**M10.** Line 282: Change “WIP” to “WPI”.

Authors reply: We corrected the abbreviation of the Widespread Pain Index from WIP to WPI throughout the manuscript.

**M11.** Line 288: Remove the first instance of “Dehghan et al.” as it doesn’t make sense to have the citation immediately followed by the full reference.

Authors reply: To improve readability we changed the footnote describing the selection of the ROI in all three tables as suggested by the reviewer.

**M12.** Line 294: Move the definition of SI to before the definition of SII.

Authors reply: As suggested by the reviewer we changed the order of definitions.

**M13.** Line 297: I suggest changing “We found no differences in rsFC among areas associated with chronic pain in fibromyalgia neither in crude not adjusted analyses after FDR correction” to “We found no differences in rsFC among areas associated with chronic pain in fibromyalgia in adjusted or unadjusted analyses after FDR correction”.

Authors reply: We adapted the sentence as suggested by the reviewer and as suggestion by the reviewer’s comment #4 use now FWE throughout the manuscript.

**M14.** Line 300: Remove the dash in “group differences”. Also on line 328.

Authors reply: As suggested by the reviewer we removed the dash in “group differences” throughout the manuscript.

**M15.** Line 305: Place “(GMD)” immediately after “grey matter density”. Also, use either “pre-defined” or “pre-specified” or “a priori” consistently when referring to the ROIs you used, not all of them.

Authors reply: As suggested by the reviewer we used the same term “pre-specified” consistently throughout the manuscript. Likewise we placed “(GMV)” immediately after grey matter volume.

**M16.** Lines 312 and 322: FDR is not used in the Tables so remove it from the footers for Tables 3 and 4 (or add FDR corrected results).

Authors reply: In line with comment #4 and #7 of the reviewer we added FWE corrected results and therefore also refrained from removing the footnote.

**M17.** Line 314: Add definition of SI.

Authors reply: We added the definition of SI to the footnote of table 3 and of SI and SII to the footnote of table 4:

**M18.** Line 327: State for what the subgroup’s analyses were adjusted.

Authors reply: We clarified the co-variables included in the model in the text as follows:

Adjusted whole-brain subgroup-analyses, including depression, anxiety, and mean global rsCBF or total intracranial volume as co-variables, of 11 (34%) patients not taking centrally acting drugs and matched controls showed no group differences in rsCBF, GMV and CoTh.

**M19.** Lines 355-6: Clarify that pain may be invisible at the individual level, but not at the group level when comparing patients to healthy controls.

Authors reply: Unfortunately we are not able to understand this reviewer’s comment. We kindly ask the reviewer to further specify if this minor comment should be addressed. We are happy to provide further information if needed.

**M20.** Line 371: add “and controls” to the end of “they did not find group differences of rsCBF between fibromyalgia patients”.

Authors reply: We adjusted the sentence as suggested by the reviewer.

*“However, they didn’t find group differences of rsCBF between fibromyalgia patients and controls, what is in line with our findings.”*

**M21.** Line 387 states that you found “robust” results, but this statement does not seem supported as most of your results were not significant.

Authors reply: We adapted the sentence as follows:

*“Hence, we adjusted all our analyses for depression and anxiety and again were unable to find any group differences.”*
